# Identifying priorities and developing collaborative action plans to improve accessible housing practice, policy, and research in Canada

**DOI:** 10.1371/journal.pone.0318458

**Published:** 2025-02-10

**Authors:** Sander L. Hitzig, Kirstin E. Yuzwa, Linda Weichel, Eva Cohen, Luke Anderson, Peter Athanasopoulos, Krista L. Best, Marco Chow, Anita Kaiser, Franca Tomasella, Sara J. T. Guilcher, Vanessa K. Noonan, Richard Joy, Anika Abdullah, Farah Bacchus-Misir, Amy Chan, Marnie Courage, Nikoletta Erdelyi, Deborah Fletcher, Siobhan Galeazzi-Stirling, Gary Gladstone, Jenn Green, Monte Hardy, Evelyn Harris, Jeffrey Kerr, Judi Lytle, Kyla MacGinnis, Gary Malkowski, David Rosenbaum, Julie Sawchuk, Mikiko Terashima, Yu-Ling Yin, Sue VanDeVelde-Coke, Christine L. Sheppard

**Affiliations:** 1 St. John’s Rehab Research Program, Sunnybrook Research Institute, Sunnybrook Health Sciences Centre, Toronto, Ontario, Canada; 2 Department of Occupational Science and Occupational Therapy, Temerty Faculty of Medicine, University of Toronto, Toronto, Ontario, Canada; 3 Weichel Consulting on behalf of the Urban Land Institute, Toronto, Ontario, Canada; 4 StopGap Foundation, Toronto, Ontario, Canada; 5 Spinal Cord Injury Ontario, Toronto, Ontario, Canada; 6 School of Rehabilitation Sciences, Universite Laval, Centre for Interdisciplinary Research in Rehabilitation and Social Integration (Cirris), CIUSSS de la Capitale-Nationale, Quebec City, QC, Canada; 7 The Daniels Corporation, Toronto, Ontario, Canada; 8 KITE Research Institute, Toronto Rehabilitation Institute-University Health Network, Toronto, ON, Canada; 9 Person with Lived Experience, Canada; 10 Leslie Dan Faculty of Pharmacy, University of Toronto, Toronto, Ontario, Canada; 11 Praxis Spinal Cord Institute, Vancouver, British Columbia, Canada; 12 Urban Land Institute–Toronto, Toronto, Ontario, Canada; 13 Parkin Architects Limited, Toronto, Ontario, Canada; 14 Choice Properties, Toronto, Ontario, Canada; 15 Incluzia, Winnipeg, Manitoba, Canada; 16 Royal LePage Estate Realty, Toronto, Ontario, Canada; 17 The Reena Foundation, Toronto, Ontario, Canada; 18 Greenwin Corporation, Toronto, Ontario, Canada; 19 Bob Rumball Canadian Centre of Excellence for the Deaf, Toronto, Ontario, Canada; 20 EllisDon, Toronto, Ontario, Canada; 21 Barrier Free Real Estate—Re/Max, Toronto, Ontario, Canada; 22 City of Burlington, Burlington, Ontario, Canada; 23 BuildAble, Ottawa, Ontario, Canada; 24 Sign Language Institute Canada | Canadian Cultural Society of the Deaf, Toronto, Ontario, Canada; 25 Sawchuk Accessible Solutions, Blyth, Ontario, Canada; 26 Department of Community Health and Epidemiology, School of Planning, Dalhousie University, Nova Scotia, Halifax, Canada; 27 The Wellesley Institute, Toronto, Ontario, Canada; 28 The Factor-Inwentash Faculty of Social Work, University of Toronto, Toronto, Ontario, Canada; 29 Kerry’s Place Autism Services, Toronto, Ontario, Canada; University of Saskatchewan, CANADA

## Abstract

This article describes the development of priorities and actions to improve the state of research, policy, and practice related to accessible housing in Canada for persons with disability or with accessible housing needs. A modified Delphi approach with an expert cross-sectoral panel was used to gain convergence on a set of priorities for advancing the accessible housing field in Canada. This included circulating an anonymous pre-meeting survey (N = 49) followed by an in-person planning meeting (N = 45). The expert panel at the in-person meeting identified three clusters of priorities from an initial list of 21 priorities, which included: 1) engaging with all levels of government to support accessible housing efforts; 2) developing educational resources to raise awareness about accessible housing, and creating services to facilitate locating and acquiring accessible housing; and 3) fostering meaningful engagement across key interest groups and sectors to find solutions to enact positive change in this space. The findings provide an initial roadmap for bringing greater cohesion to the accessible housing field, which will enable cross-sectoral partnerships and collective action towards informing the next generation of accessible housing standards, regulations and practices for people with accessible housing needs.

## Introduction

Access to appropriate and affordable housing is a human right and is critical for promoting health, dignity, safety, inclusion, and community participation [1]. In Canada, people with disability and others with accessible housing needs, such as those in the Deaf, Hard of Hearing, Deafblind, Sight-loss, and Neurodivergent communities, whose members do not necessarily identify with having a disability or being disabled, struggle with housing [2], and this issue is expected to grow over time. The number of people in Canada with one or more disabilities limiting participation in daily activities has increased by 5% over the past 5 years, such that it is now 27% of the population, or 8 million people [2]. As defined in Canadian legislation, a disability is any impairment that is permanent, temporary, or episodic, which leads to a significant limit on an individual’s ability to carry out some of life’s important functions or activities [3]. According to the social model of disability, a person’s disability can be considered to arise from societal barriers, rather than their physical or mental impairments [4]. For example, a home that is inaccessible may be considered disabling since it can prevent a person from being able to move throughout their environment and from being able to complete self-care (using the bathroom, bathing or showering), or home-care (house cleaning, cooking, laundry) activities, which are tasks they may otherwise be able to do or could perform with acceptable efficiency and independence in an accessible home. This inaccessibility can worsen a person’s health and well-being and the home environment itself may be considered to “entrench” the disability [5].

Canadians with a disability have been identified as a vulnerable group for not having their core housing needs met [6]. Core housing needs include whether a household is affordable (paying less than 30% of income on shelter costs), suitable (enough space for the household composition), and adequate (housing in good repair). The difficulties experienced by the disability community in finding appropriate housing is not surprising given the high rates of low-income reported in people with disabilities, long-standing issues of discrimination, and added expenses associated with having a disability, such as accessibility-related home modifications [2, 7–9]. Consequently, many people with disabilities have unmet housing needs (i.e., need for widened doors, lack of a roll-in shower, ramps), which results in them living inaccessible homes [10]. In extreme cases where accessible housing cannot be found, some people with a disability end up living within very limited areas in their home, living with their family (e.g., parents, adult children), or in institutional settings (i.e., long-term care), which are often considered as last options [11–13].

The prioritization of accessible housing in Canada is hampered by a weak evidence base, due to both a lack of studies and poor to moderate quality evidence [9, 14], in addition to a national disconnect across sectors (i.e., academia, practitioners, policy-makers, etc.). However, there are emerging pockets of legislative, academic, disability advocacy and housing industry silos of activity, which are starting to converge towards collective action. Some evidence of this activity includes the release of the CSA/ASC B652:23 Accessible dwellings standard [15], recent academic research reviewing accessible housing and related national policies [16, 17], and the formation of cross-sectoral and cross-disability coalitions focused on advancing accessible housing [18].

Recognizing the increasing need for accessible housing to support Canada’s growing number of persons with disabilities and aging population, our team organized a planning event to collaboratively identify and obtain consensus on a set of priorities in accessible housing. The intended outcomes of this event were to build greater cohesion in the field, stimulate collective action, and inform the next generation of accessible housing standards, regulations and practices for people with accessible housing needs.

## Material and methods

**Fig 1:**
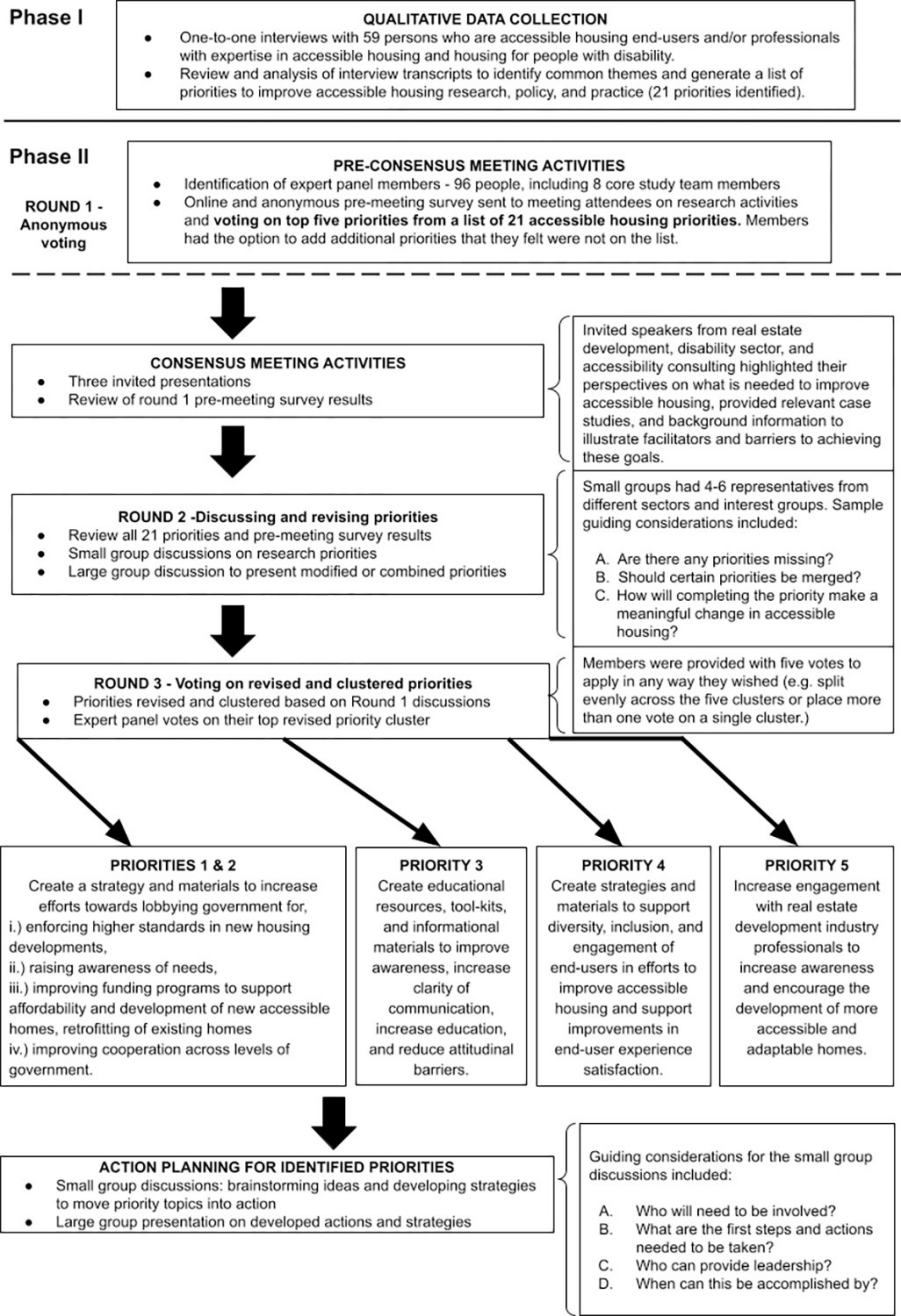
Modified Delphi approach and consensus workshop structure used to generate the top priorities for improving accessible housing.

Our team used a modified Delphi approach to establish Canadian accessible housing priorities. A Delphi method is a “structured group communication method for soliciting expert opinion about complex problems or novel ideas, through a series of surveys and controlled feedback” [19]. The Delphi approach traditionally does not have participants interact with one another, rather, it use a series of surveys to obtain anonymous feedback from participants to achieve consensus [20]. For this accessible housing meeting, we modified the approach by using three total rounds, which consisted of an anonymous survey (round 1) followed by two rounds of non-anonymous face-to-face interactions via an expert panel. Our team has successfully used a similarly modified Delphi approach in the past to co-create shared priorities from diverse key interest groups [21, 22]. In this approach, round 2 consisted of in-person discussions that were more divergent tone while panelists critically reflected on results of the anonymous voting on the broad list of potential priorities. These discussions were followed by round 3, which used a two-step convergent process to further develop and select the top priorities (**Fig 1**). Our team used a neutral facilitator to manage rounds 2 and 3, which took place at the in-person event. All phases and activities undertaken for this event received approval from the research ethics board at the Sunnybrook Health Sciences Centre (REB# 5775).

### Expert panel identification and accessibility considerations

An essential phase of a Delphi process is the selection of an expert panel, given the input provided by participating members will have a direct impact on the quality of the produced results [20]. To achieve this goal, study team members (SLH, KEY, LW, CLS, LA, MC, PA, MC) undertook an environmental scan of persons working in the field across Canada and leveraged our existing networks of professionals across the housing, disability, and healthcare sectors to enable diverse perspectives on accessible housing. We also worked with our network to identify persons with lived experience of having accessible housing needs and advocates for various end-user groups to participate in this process, which included persons with physical disabilities, persons from the Deaf and Sight-loss communities, and community representatives for persons with neurodivergence including autism, developmental, and intellectual disabilities.

To enhance participation and engagement, we ensured a physically accessible venue, provided digitally accessible materials in advance to attendees, and asked speakers to describe visual content in their presentations. Additionally, we offered American Sign Language interpretation, real-time captioning, and personal support worker services for assistance with personal items, clothing, bathroom needs, and food or beverages.

### Stage 1 –initial identification of priorities

The initial set of priorities used for the anonymous round 1 voting were generated by study team members (SLH, KEY, EC, FBM, SGS) through obtaining and reviewing interview and focus group data with 59 individuals on accessible housing issues [23]. Interview data were collected from 21 persons with accessible housing needs and concerns (e.g., physical disability(ies), deafness, sight-loss), as well as from 25 professionals working across different sectors relevant to accessible housing, including for-profit and not-for-profit real estate builders, accessible housing consultants, advocacy groups, healthcare professionals, and legal professionals. Recruitment and interview data were collected between July 1, 2023 until November 1, 2023. Thirteen participants were a person with lived experience of requiring accessible housing who also worked in a professional capacity related to disability or housing. Participants in the qualitative interviews were primarily residents of Ontario, Canada (n = 58) with one being a resident of Manitoba, Canada (n = 1).

A qualitative descriptive inductive approach [24] was used to collect data from participants about their experiences of obtaining or providing accessible housing, which included questions related to existing building codes, regulations and laws that impeded or facilitated accessible housing, and to obtain perspectives on what was needed to inform the next generation of accessible housing standards. Interview data was coded by members of the study team (SLH, KY, SGS, FBM, EC). Four coders independently coded identical transcripts from each participant group to assess inter-coder reliability. Conflicts were resolved through team dialogue focusing on shared meaning. This iterative process resulted in coding framework, which all coders applied when independently analyzing the remaining transcripts. This process yielded a list of 21 priorities (**Table 1**), which broadly encompassed issues on: 1) legislation and regulation; 2) policy and financial incentives; 3) marketing and communication; and 4) education, resources and capacity building. The topics and generated priorities were reviewed by members of the study team (LW, MC, CLS, FT, LA), which included members with expertise in real estate development, accessible housing, research, and lived experience of disability. A full report on the qualitative data will be reported elsewhere.

**Table 1:** List of priorities and rankings.

Priority Description	Ranking
Strategically engage with all levels of government to improve awareness of needs and cooperation across levels of government	22
Increase efforts to strategically engage with the real estate developers to help support creation of more adaptable housing	22
Lobby government to improve regulation and enforcement a higher accessibility standards for housing	20
Improve the quality and quantity of information on the availability of accessible homes in the current housing stock through multiple listing service (MSL) and other similar platforms or services	17
Increase efforts to improve public awareness of the benefits and universal need for accessible homes	16
Map and disseminate currently available funding and financing resources for building and modifying accessible homes	15
Create and disseminate an inventory of accessible housing needs and priorities not captured by technical documents	14
Develop and advocate for a set of standard terminology and definitions on accessible housing	12
Create a strategy to support and enable meaningful engagement of people with disability(ies) with real estate developers	11
Increase efforts to create materials and resources that support capacity-building on accessibility for existing housing professionals	11
Increase efforts towards enhancing the availability, quality and access to supportive housing services	11
Work to improve resident safety through incorporation of accessibility features in common spaces in multi-unit buildings	10
Increase capacity-building efforts geared towards the next generation of accessible housing professionals	9
Develop materials to support the need for creation of new accessible homes as opposed to retro-fitting existing homes	9
Enhance research efforts to improve knowledge about accessible housing and demonstrate impact of accessible housing	8
Enhance efforts to support inclusivity and diversity when engaging people in collective efforts to improve accessible housing	6
Increase efforts to support the availability and access for people with disability(ies) to have supportive in-home technologies	5
Improve marketing efforts to the broader public that focus on aesthetic appeal and desirability of accessible design features	4
Map international policies, programs related to accessible housing to help to inform potential changes in Canada	4
Develop a strategy on how to capture and disseminate data on the total accessible housing stock in Canada	3
Increase awareness and availability of resources on the rights and legal protections around accessible housing in Canada	2
**Additional Priorities**	
Create and promote housing designs with built-in closets, shelving, and storage to ease affordability and independence when moving homes	
Create and promote housing designs that prioritize autonomy, safety, and independent living via ease of cleaning and hazard reduction	
Increase lobbying efforts to government for improved funding of new accessible housing and accessibility related home modifications	

### Pre-meeting activities and expert speakers

It is important the expert panel is provided with sufficient context and background information to move through the Delphi rounds [25]. To achieve this goal, our team created an online survey using the LIME Surveys platform to request invited event panelists to review and select their top five priorities. Invitees were also encouraged to add their own priorities if those listed did not capture what they felt was important to improve accessible housing in Canada. This survey also contained optional self-identification questions to track and assess the diversity and inclusion of historically under-represented communities in our research sample. The survey data was collected between December 12, 2023 until January 15, 2024. Five days prior to the meeting, which was held on February 2, 2024, we provided invitees a meeting booklet summarizing the intended objectives of the consensus event, the pre-meeting survey results, and a high-level summary of the qualitative interview findings that informed the generation of the initial 21 priorities.

At the consensus event, three experts shared perspectives on accessible housing: two individuals with lived disability experience—one focusing on advocacy and policy, and another, a certified accessibility consultant, discussing housing’s health impacts—and a real estate developer providing a market perspective. Additionally, pre-meeting survey results and additional participant identified priorities from the first Delphi round were presented.

## Results and discussion

### Round 1—pre-meeting survey results

Eighty-two people received an invitation to participate in the meeting and online survey, including 10 study team members who reviewed study materials but did not participate in data collection or analysis to derive the list of priorities (MC, RJ, VKN, AK, SJTG, PA, LA, FT, KLB, CLS). Forty-nine invitees anonymously completed the pre-meeting survey and round 1 voting (**Table 1**). Study team members directly involved in data collection and analysis (SLH, KEY, LW, EC, FBM, SGS) were not included in the list of invitees, and did not vote during the Delphi activities to avoid potential bias. The response rate was impacted by invited experts who did not respond to the email invitation (16/82 invitees) and those who could not attend the event but whom identified a suitable alternate (8/82invitees), such that an additional 8 participants received an invitation. Survey respondents represented a variety of sectors and professional roles, and 37% of respondents identified as a person with a disability (**Table 2**). With further respect to diversity and inclusion, 33% of respondents identified being from an equity-deserving racial or ethnic group, 18% identified as someone from the 2SLGBTQIA+ community, and 65% identified as women.

**Table 2:** Pre-meeting survey respondents’ profiles (N = 49).

Role–some respondents identified having more than one role	(n)
Housing professional (e.g., developer, architect, realtor, designer, contractor, accessibility consultant, etc.)	22
Person with lived experience of disability(ies)	18
Advocacy or non-governmental agency professional	10
Researcher (e.g., university faculty or staff, research institute scientist or staff, etc.)	10
Healthcare professional (e.g. social worker, occupational therapist, physician etc.)	5
**Gender Identity**	**(n)**
Woman	31
Man	16
Intersex, Transgender man or woman, Non-binary, Gender-fluid, Two-spirit or other	1
Not reported	1
**Sexual Orientation**	**(n)**
Heterosexual	37
2SLGBTQIA+ (Asexual, Bisexual, Gay, Lesbian, Pansexual, Queer, Two-spirit, or other)	8
Not reported	4
**Ethnic or Racial Heritage**	**(n)**
White/Caucasian (North American or European)	32
Other race and ethnicities (East Asian, South Asian, Black, Jewish, Mixed heritage)	16
Not reported	1

The top priorities from the Round 1 voting (**Table 1**) were: 1) creating a strategy to engage with government to and improve awareness of needs, and improve cooperation across all levels of government with respect to advancing accessible housing outcomes, and 2) creating a strategy to facilitate the real estate development industry to build more housing that is adaptable. Adaptable housing is built in such a way that can more easily and affordably be modified to incorporate accessible or universal design features in the future (e.g., widened doorframes and corridors, wall reinforcements to support grab bar installation, and plumbing rough-ins that allow knee clearance for wheelchair users, etc.) [17]. The priority receiving the third highest number of votes was, increasing efforts to engage with the government to improve enforcement and regulation of higher accessibility standards for new housing developments, particularly when government financing is used to support the development. Survey respondents added three new priorities in addition to the original 21 generated from the qualitative interviews.

### Rounds 2 and 3—consensus workshop results

Forty-five people attended the in-person consensus event in Toronto (Ontario), including the ten study team members (**Table 3)**. Of the forty-five attendees, 82% (37/45) completed all three rounds of Delphi activities. Four attendees did not participate in the Delphi rounds outside of facilitating and taking notes during the rounds 2 and 3 small group discussions because they were study team members involved in data collection (SLH, KEY, EC, LW). Four invitees did not complete the round 1 survey voting but did attend the in-person event to participate in rounds 2 and 3. A majority of panelists were from across Ontario with panelists also attending from other major Canadian cities in the provinces of British Columbia (n = 1), Manitoba (n = 1), Quebec (n = 1) and Nova Scotia (n = 1). A neutral facilitator led the meeting, whose role was to provide direction and mediation during the small group discussions, synthesize group feedback, and guide panelists towards consensus of priority areas. To help further minimize bias, members of the core study team were pre-assigned to one of the nine round table groups, each comprised of four to six panelists from different sectors and backgrounds (ex. a market housing developer, NGO accessible housing service provider, researcher, real estate agent, and end-user with relevant lived experience, etc.). Each of the nine round tables had a pre-assigned facilitator who took notes on a large visible notepad used to help guide the discussions.

**Table 3:** Consensus meeting attendee roles (N = 45).

**Role–some respondents identified having more than one role; eight were core study team members**	**Total**
Housing professional (e.g., developer, architect, realtor, designer, contractor, accessibility consultant, etc.)	17
Person with lived experience of disability(ies)	15
Advocacy or non-governmental agency professional	11
Researcher (e.g., university faculty or staff, research institute scientist or staff, etc.)	9
Healthcare professional (e.g. social worker, occupational therapist, physician etc.)	3
Policy-maker (e.g., municipal, provincial or federal government employee or elected official)	2
Family caregiver for a person with disability(ies)	1
**Gender Identity**	**Total**
Woman	25
Man	19
Intersex, Transgender man or woman, Non-binary, Gender-fluid, Two-spirit or other	1

After the round 1 pre-meeting survey voting results were presented, panelists were asked to critically reflect on the priorities, revise or merge priorities where they felt it was necessary, and to discuss which priorities were the most important to pursue action on over the next 1–3 years. A period of 1–3 years was selected as it presented a realistic length of time in which significant changes to policy, practice and research in the accessible housing field could be realized. During this process, panelists wrote down their top priorities, which in some cases included merging priorities from the original list and modifying existing priorities to offer a more nuanced focus on the topic. Each of the nine groups then provided a high level summary to the larger group of panelists about which priorities their table felt were the most important to pursue.

Once completed, the neutral facilitator and the core study team worked to organize the small group priorities into clusters based on their commonalities. Any original priorities from the full list that were not discussed or that were identified as important in the small group sessions were removed and archived. The revised and clustered priorities were then posted around the physical space, and panelists were provided five stickers to vote on their preferred priority. Panelists were informed they could spread their five votes in any way they desired, which could include putting one or more votes on any given priority. This method was chosen as a result of its successful execution in prior consensus building efforts [21, 22].

#### Identified accessible housing priorities

The resulting five clustered priorities (see **Fig 1**) were: 1) increase efforts to lobby the government to create mechanisms for enforcement of higher accessibility standards for housing; 2) create a strategy on how to engage all levels of government–improve cooperation, raise awareness of needs and priorities, and improve funding; 3) create and disseminate educational materials and resources to fill knowledge gaps, and to improve efficiency of matching people with available accessible properties; 4) create a strategy of inclusivity and engagement that supports people from different disability groups, ages, and lived experiences to be included in efforts to improve accessible housing, and ways to support improving end-user satisfaction with the process and result of having accessible home needs met; and 5) create a strategy, resources, and materials to engage with the real estate development industry to support and increase their efforts towards building more new accessible and adaptable homes.

Generation of these five priorities for the third round in the Delphi process was challenging given the complexity and breadth of the topic area, and the intersectional and interdependent nature of the priorities. These factors resulted in study team members (KEY, SLH, KLB, EC) needing to identify common and key elements of focus from the small group discussions and merging priorities into larger priority domains such that they better reflected results from the first two Delphi rounds and small group discussions. Despite this challenge, there was general support amongst panelists of the resulting identified priority statements presented for Round 3 voting and no expert panel members objected to focusing discussions on the five top domains presented for further development.

### Prioritized areas for advancement and identifying strategies

Following the third Delphi round involving in-person voting, each of the top five identified priorities were assigned to one or two round tables, and panelists were invited to choose a priority domain they wished to further discuss. This round of small group discussions focused on strategic action planning for advancement of the priority area. Each table had a designated note-taker who helped focus and track the discussion using guiding questions centered on creating strategies for priority advancement over the next one-three years.

#### Identified priority: Government engagement

For this report, we collated the two top priority clusters related to government engagement since they both overlapped with respect to: 1) lobbying for increased and improved funding programs to support building new accessible homes and accessibility related modification of existing homes; and 2) the enforcement of higher accessibility standards (Fig 1). To achieve this goal, panelists stressed the need to raise awareness about the importance of accessible housing across all levels of government (municipal, provincial, federal) as well as to develop a strategy to improve coordination across all levels to advance outcomes.

To increase the critically low stock of accessible homes in Canada, participants emphasized the need for targeted funding programs. These programs should support both new housing developments (market and non-market, including supportive housing) and accessibility modifications for existing single-family and multi-unit residences. Suggested measures included expanding financial incentives, enhancing funding under Canada’s National Housing Strategy [6], introducing minimum occupancy-based funding, and broadening tax rebate programs. Panelists noted that inconsistent and frequently changing programs make it difficult for developers to secure funding, hindering accessible housing projects. A proposed solution is a stable, long-term funding model to incentivize developers to prioritize accessibility. Additionally, programs are needed to help individuals with accessibility needs secure suitable housing.

In particular, panelists generally considered developing policies that support subsidized procurement of building materials needed to create accessible home features to have the potential to motivate developers and the public to build or modify homes to support accessibility. Panelists also frequently cited increased costs associated with purchasing construction materials as a major barrier limiting accessible housing development.

A theme frequently considered by panelists was the urgent need for the inclusion and enforcement of higher accessibility standards in all new housing supported by government funds, particularly given current initiatives to rapidly increase the housing stock in Canada through programs such as the federal National Housing Strategy Housing Accelerator Fund [26]. Panelists discussed the need for policy and regulation that prioritizes accessibility in government funded housing, such that all new housing development projects supported by government funds should minimally have the most commonly needed key accessibility features, such as barrier-free entrances, widened doorways and corridors, and plywood reinforcement on bathroom walls to support the addition of grab bars. Overall, panelists expressed that the current Canadian system was overly reliant on incentives and goodwill rather than enforcement.

To mobilize meaningful change in how policies, regulations, and standards are applied in Canada to promote accessible housing, the key strategy suggested by panelists was to form more robust national level cross-sectoral partnerships among key interest groups focused on improving accessible housing to create a unified mechanism to educate and lobby all levels of government on this issue. One suggested approach was to obtain academic funding to bring diverse groups together to support action planning. As a short-term goal, this would include mapping and targeting existing regulations and policies, creating a collective statement with concrete recommendations, and then mobilizing this information into action through lobbying for immediate change given the urgency in need for action.

A long-term goal identified by panelists was to develop new and improved legislation that mandates higher levels of accessibility in all housing, including changes to the National Building Code of Canada. As well, panelists expressed there was need for greater cooperation across levels of governments to both incentivize accessible housing and institute stricter and improved enforcement. Strategies suggested by panelists to help achieve this goal were having provinces mandate that municipalities have an accessibility consultant whose role is to review housing development proposals and support the enforcement of accessibility standards, with punitive actions if required (e.g., shutting down a development). There were several insights, considerations and recommendations that all the panelists discussed related to engaging with the government, which are summarized in **S1 Table**.

*Identified priority*: *Accessible housing educational resources and services*. The expert panel highlighted the need to create and disseminate educational materials and resources to fill knowledge gaps on accessible housing. This included providing information related to available support services that focus on accessibility housing, processes for building and designing accessible homes, collating information on financing and funding programs, and general public awareness of the universal need for and benefits of accessible housing.

Panelists felt the creation of a central hub or portal with educational resources and tool-kits to support a variety of end-users (e.g., real estate developers, non-governmental organization (NGOs), policy makers, etc.), where information and research on accessible housing could be found. To achieve this goal, some strategies put forth were to establish funding (e.g., academic) and sponsorship (e.g., industry), as well as to create a clear leadership structure regarding the management of this accessible housing knowledge portal. With the understanding that many services and some funding programs are offered at a municipal level, panelists felt the short-term goal would be to pilot test this type of portal at a local level before engaging in a long-term goal of establishing larger provincial and national directories of accessible housing portals.

With regard to content domains, panelists highlighted the importance of resources geared specifically towards housing developers and the need to create new core competencies and improved curriculums in post-secondary institutions for home building tradespeople, architects, engineers, etc. Panelists suggested that tailored resources could simplify design standards and regulations, provide options for funding and partnership, and dispel common misconceptions around the costs, aesthetics, and legality of building above current minimum building code standards to support improvements in accessibility. To change attitudes and beliefs regarding accessible housing, panelists suggested including a “why” component in curriculum and education efforts. Meaning, knowledge should be provided that stresses the importance of building for the future to support aging in the right place, which could be achieved by improving opportunities for inclusive social engagement, using participatory approaches that showcase enabling and disabling home environments for people, and the discussion of universal benefits of accessible housing for the general population.

In particular, panelists noted the importance of providing clarity on how building to minimum building code standards may fall short towards meeting individuals’ rights to housing as outlined in international, national and provincial human rights codes and legislation (e.g., UN Convention on the Rights of Persons with Disabilities, the Canadian Charter of Rights and Freedoms). Therefore, finding ways to have real estate developers incorporate universal design principles, and at a minimum, visitable design standards, should become standard practice in all housing design and development projects. [15, 27, 28]. Visitable design denotes three accessibility features required for people with mobility impairments to be able to visit a home, 1) a zero-step level entry, 2) all main floor interior doors having a clear opening width of 32–36 in., and 3) at least one half-bathroom, but preferably a full bathroom on the main floor with a 60-in. turning circle [15].

Panelists also discussed the need to increase the availability of services and to improve marketing of accessible properties in the current housing stock, which could make it easier for people to find accessible properties. Panelists with disabilities identified this need, which emerged from their own experiences of being unable to find available accessible homes since the accessibility features in real estate listings typically lacked the necessary details regarding the home’s accessibility features. As well, people with sight-loss expressed challenges with a lack of support services available to help them to search for and assess potential properties both online and on-site. Panelists noted that a single checkbox indicating a property is wheelchair accessible is not sufficient to determine the accessibility features present in the home, and that more detailed specifications are required to understand whether features of the property match an individual’s accessibility related needs. Other valuable information required to make the determination of the suitability of the home includes, but is not limited to, the types and widths of entryways, exits, and doorways, specific bathroom and kitchen features, and floorplans. As well, panelists noted that developers or rental unit managers expressed difficulty with efficiently finding appropriate buyers or tenants to fill accessible property vacancies. In some cases, housing developers reported that their accessible units were being rented or sold to people without explicit accessibility needs due to time-based and financial pressures to fill a unit.

A strategy discussed to address this issue was to create better metrics, practices, and services that could help people with accessible housing needs to find homes that would match their needs. This could be modeled from existing services currently being offered in parts of Canada, such as The Right Fit in Vancouver (British Columbia), which is a multi-partner service designed to address the crisis in wheelchair accessible housing by matching affordable, accessible homes and independent living supports. Other suggested mechanisms by panelists to enhance the matching of appropriate homes included having a broad, government supported expansion of focused support services for matching individuals with available accessible properties in both urban and rural locations. To achieve this goal, panelists recommended exploring the use of innovative technologies to help with the matching process, and improved property marketing services for the general population that provide greater detail on accessible features within properties and needed services and features in surrounding neighborhoods (i.e., improving accessibility-related information on multiple listing services [MLS]).

*Identified priority*: *Diversity*, *inclusion*, *and engagement strategies*. A cross-cutting issue when discussing priorities for advancing accessible housing was the need for meaningful engagement across sectors. This included finding ways to facilitate the inclusion and engagement of diverse people with lived experience who have accessible housing needs. Some panelists expressed that accessible housing has been typically conceptualized as being for wheelchair-users and that the voices and needs of other populations, such as those from the Deaf, Hard of Hearing, Deafblind, Sight-loss, and Neurodivergent communities, have been left out, resulting in safety, health, and well-being concerns. Examples of reported needs for these populations include: 1) entryway intercoms, emergency announcements, and elevator communication systems designed for individuals with deafness or sight loss, 2) visual alerts on carbon monoxide detectors for safety among people with deafness, 3) under-cabinet lighting and improved electrical infrastructure to support diverse lighting needs for individuals with sight loss, 4) interior soundproofing to aid neurodivergent individuals with auditory sensitivities, and 5) shortened travel paths, low-rise stairs and curbs, and increased seating access for ambulatory individuals with limited mobility, including many older adults. These issues are rarely addressed in existing literature. Further research is needed to identify needs across historically under-represented communities to better inform future housing design standards and strategies.

Panelists noted that to promote inclusivity, there is a need to find ways to meaningfully engage with the real estate development field, as with other sectors working in this space (e.g., policy, academia, etc.). Additionally discussed was a need to reduce attitudinal and societal barriers related to accessible housing across sectors, which was considered to include de-medicalizing accessibility and supporting efforts to showcase the possibilities for creative design and esthetic appeal of home accessibility features. One suggested approach was to encourage developers to increase diversity and representation of people with disabilities in their hiring practices. Panelists discussed that this would generate opportunities for meaningful engagement with the real estate development field, as well as other involved sectors, and would lead to the creation of educational content and training programs on accessible housing, and other inclusive practices (e.g., providing digital and print materials related to housing in accessible formats).

Panelists emphasized the importance of involving accessible housing end-users in developing and delivering post-secondary curricula for students pursuing careers in housing development, design, and construction (e.g., trade schools, architecture, interior design). Conversely, end-user representatives highlighted the value of understanding the challenges housing developers and designers face in supporting accessible housing. This mutual exchange could foster dialogue and generate effective strategies to make accessible housing more appealing for developers to prioritize.

In general, panelists identified there is a need to create opportunities for allyship, including the creation of leadership roles for accessible housing end-users. This may facilitate the use of inclusive language, advocacy for consumer protections, as well as changing perceptions such that accessible housing is viewed as a shared social responsibility.

## Discussion

The present initiative describes efforts to establish consensus on accessible housing priorities in Canada from multi-disciplinary key interest groups (e.g., policy, practice, research, advocacy, lived experience, etc.). The outcomes of this process revealed several issues requiring attention to advance the accessible housing field, and that consensus on specific and well-defined priorities was challenging to achieve. Although the convened expert panel generally was supportive of the identified priorities at the in-person event, it appears our team obtained convergence, rather than consensus, on three broad priority areas with different sub-priorities requiring action. These included: 1) engagement with government to support accessible housing efforts; 2) developing educational resources to raise awareness about accessible housing, and to create services to make it easier for people to locate and acquire housing; and 3) fostering meaningful engagement across key interest groups and sectors to find solutions to enact positive change in this space.

From our perspective, a starting point for addressing the identified clusters of priorities is to clarify existing terminology and concepts used to describe accessible housing. In Canada, there are several different terms and standards used interchangeably across sectors for accessibility in housing, such as, barrier-free, visitable, adaptable, universal design, and accessible. These terms may refer to the degree to which a home is accessible, a concept or theory related to accessibility, or in some cases, a locally developed, municipal, provincial, or national building standards aimed at achieving these concepts or levels of accessibility in practice. The interchangeable use of terminology unfortunately creates problems with miscommunication given the terms have quite different implicit meanings across the individuals and groups using them. Hence, one recommendation that could advance improvements related to the identified priorities is to promote the use of clear, consistent, and widely adopted language to describe accessible housing.

An enhanced consistency in interpretation, practice application, and enforcement of accessible housing standards across jurisdictions and levels of government would lead to improvements in: 1) communication between actors in key interest groups who work across the housing, healthcare, disability, and policy sectors; 2) clarity in interpretation and implementation of accessible housing research to improve study quality and knowledge translation into practice; and; 3) ease of interpretation of cross-jurisdictional law with respect to cases of discrimination and enforcement of human rights in relation to accessible housing [9, 29].

Other countries that have similar governance to Canada have created and adopted more robust and clear terminology, which is beginning to lead to advancements in policy and broad adoption of higher standards to improve accessible housing outcomes. For example, the United Kingdom has national design standards for three tiers of accessible housing designated as M4(1) Visible dwellings, M4(2) Accessible and adaptable dwellings, and M4(3) Wheelchair user dwellings [30], while the Australian government has nationally adopted the Silver, Gold, and Platinum levels of their Livable Design Standards [31]. Notably, the Silver standard (which is similar in scope to the visitable and adaptable concepts in Canada) was broadly adopted into the Australian National Building Code in 2023 [32]. Notably, the design standards in Australia, (silver, gold, platinum) do not include the name of a specific concept related to accessibility and in the United Kingdom, the design standards offer a name (i.e., M4-1, M4-2, M4-3) in addition to the accessibility concept which they are geared towards achieving. There is a need, however, to critically reflect on whether similarly structured hierarchical tiers of accessibility standards can adequately meet the need to recognize, support, and protect individuals’ rights to adequate housing, and whether current terminologies used in Canada are sufficient or whether more radical change is needed to drive advancements. Regardless, exploring how other similar countries to Canada have made larger strides in the field could inform ways to elevate the adoption (and mandating of) elements within Canada’s voluntary standards (Accessible dwellings standard [CSA/ASC B652] [15]) into current jurisdictional building codes used across our country, and into the future harmonized national building code of Canada.

The present initiative has established a broad roadmap to help bring greater cohesion across sectors working to improve accessible housing in Canada. However, we acknowledge there are limitations to how our team identified priorities. Although the Delphi approach was appropriate, a challenge of our modified method was convening an in-person expert panel with diverse stakeholders from various sectors (e.g., research, real estate and housing development, disability advocacy). While their unique perspectives, shaped by professional roles and lived experiences, enriched discussions, it also led to some communication challenges throughout discussions. Adopting unified language, terminology, and national standards for accessible housing could help mitigate these communication challenges in the future and be used to guide cross-sectoral discussions in this field. Despite this challenge, other studies using Delphi methods have suggested heterogeneity amongst the expert panelists is an important factor in producing reliable results [33], and given the cross-sectoral nature of issues related to accessible housing and its population impacts, it is particularly important to promote diversity and inclusion efforts broadly in this field.

A limitation to this work is that the in-person discussions on priorities, combined with presentations by invited speakers, may have influenced the group’s decision-making. Such influences are typically minimized in traditional Delphi studies [20]. To address potential group dynamics and power imbalances in the modified Delphi process used, our team used an initial anonymous pre-meeting survey for voting and a neutral facilitator during the event. Moreover, while most participants completed all three rounds of the modified Delphi process, some individuals took part only in round 1, the online survey, or others only attended rounds 2 and 3 at the in-person event. This partial participation limited the influence that some participants had on the entirety of the decision-making process. While consideration of the ideal size of an expert panel for the Delphi method varies [33], others have noted that management of larger groups, such as that used in our study, can be challenging and may have lower response rates in part due to the need for experts to convene and spend a large block of time to provide their input [25, 33]. Despite these limitations, the convergence on identified priorities reflects the collective opinions of the group and should be interpreted accordingly [34].

Finally, our team undertook efforts to engage individuals from outside of Ontario or who represented national organizations to attend the event, but we acknowledge our initiative was Ontario centric and further work is required to ensure the identified priorities align with other provinces and territories in the country. Notably, the identified priorities broadly align with the 2023 Report from the Chief Accessibility Officer (e.g., providing training to the private sector, working with people with lived experience to raise awareness about accessibility, and the need for enforcement) [35].

Despite these limitations, there is clearly a need for action in Canada to improve the ability of people to obtain housing that meets their respective needs. Accessible housing provides a wide range of benefits to physical, mental and social wellbeing for people with disabilities [16]. For instance, having adequate accessibility in a home reduces the likelihood of requiring caregiver assistance which could help provide an economic return on investments in accessible housing [36, 37], makes people more likely to perceive feeling safer when completing daily activities [36], and enables social and leisure participation [38]. Importantly, current policies and regulations are not sufficient in protecting people’s rights to accessible housing. This is evident by the fact that disability (or those with accessible housing needs) often face barriers to obtaining housing and sustaining stable residency [1]. Many landlords discriminate on the basis of disability [39], which has included evicting people because of disability-related behaviours and failure to accommodate disability-related needs. For instance, people with sight loss face discrimination to renting apartments because of their guide dogs [40]. Similar discriminatory practices have been found to occur in people who are deaf, who are wheelchair users, or who have an intellectual disability [41, 42]. Hence, as the population ages and the rates of disability increase in Canada [2], there is a pressing need to ensure there will be sufficient affordable, adequate, and accessible housing for anyone who requires it. Doing so will reflect the values that Canada aspires to, which is a just and equitable society that acknowledges and applies practices that reflect housing as a human right [1, 43].

## Conclusion

Accessible housing is a critical issue for the disability community and those with accessible housing needs. The Canadian field has several pockets of emerging multi-sector partnerships (e.g., Accelerating Accessibility Coalition supported by the Urban Land Institute (ULI) Toronto [18]), but requires a national structure that could support different respective agendas across key interest groups towards collective action. Through a qualitative exploration followed by a modified Delphi process, we developed a set of priorities to advance accessible housing practice, policy, and research. The outcomes of this work may provide the required foundation to support on-going dialogue towards achieving these advancements, and has likely served as an important catalyst of bringing greater cohesion in the accessible housing field.

## Supporting information

S1 Table Government relations issues, proposed action(s), and potential outcome(s). (DOCX)

## References

[1] Alzheimer Society of Canada, ARCH Disability Law Centre, Canadian Association for Community Living, Canadian Mental Health Association–Toronto Branch, Council of Canadians with Disabilities, IRIS–Institute for Research and Development on Inclusion and Society, et al. Meeting Canada’s Obligations to Affordable Housing and Supports for People with Disabilities to Live Independently in the Community: Under Articles 19 and 28, Convention on the Rights of Persons with Disabilities And under Articles 2 and 11, International Covenant on Economic, Social and Cultural Rights. [posted 2017 15 May 15; cited 2024 April 17]. Available from: https://www.ohchr.org/sites/default/files/Documents/Issues/Housing/Disabilities/CivilSociety/Canada-ARCHDisabilityLawCenter.pdf

[2] Statistics Canada. Canadian Survey on Disability, 2017 to 2022. [posted 2023 December 12; cited 2024 March 17]. Available from: https://www150.statcan.gc.ca/n1/daily-quotidien/231201/dq231201b-eng.htm

[3] Government of Canada. Accessible Canada Act (S.C. 2019, c. 10). [posted 2024 April 16; cited 2024 March 17]. Available from: https://laws-lois.justice.gc.ca/eng/acts/a-0.6/

[4] Goering S. Rethinking disability: the social model of disability and chronic disease. Curr Rev Musculoskelet Med, 2015;8(2):134–138. doi: 10.1007/s12178-015-9273-z 25862485 PMC4596173

[5] Wiesel I. Lived experience and social, health and economic impacts of inaccessible housing. Report submitted to the Australian Building Codes Board RIS. University of Melbourne. [posted 2020 August 31; cited 2024 March 18]. Available from: https://disability.unimelb.edu.au/__data/assets/pdf_file/0011/3492686/RIA-Report-Survey-Findings.pdf

[6] Government of Canada. Canada’s National Housing Strategy. 2018. [cited 2024 April 11]. Available from: https://www.placetocallhome.ca/about-national-housing-strategy

[7] Gladman M, Dharamshi C, Zinman L. Economic burden of amyotrophic lateral sclerosis: a Canadian study of out-of-pocket expenses. Amyotroph Lateral Scler Frontotemporal Degener. 2014;15(5–6):426–432. doi: 10.3109/21678421.2014.932382 25025935

[8] Mattie JL, Borisoff J, Leland D, Miller WC. Development of an integrated staircase lift for home access. J Rehabil Assist Technol Eng. 2015;2: 2055668315594076. doi: 10.1177/2055668315594076 26793318 PMC4716830

[9] Reid L. Issues for Persons with Disabilities: Security of Tenure in Canada. Report submitted to the Office of the Federal Housing Advocate (OFHA). Editor, Canadian Human Rights Commission. 2022:1–36. [cited 2024 March 19]. Available at: https://homelesshub.ca/sites/default/files/attachments/Reid-issues_for_persons_with_disabilities-security_of_tenure.pdf

[10] Giesbrecht EM, Smith EM, Mortenson WB, Miller WC. Needs for mobility devices, home modifications and personal assistance among Canadians with disabilities. Health Rep, 2017;28(8):9–15. 29044443

[11] Bergmark BA, Winograd CH, Koopman C. Residence and quality of life determinants for adults with tetraplegia of traumatic spinal cord injury etiology. Spinal Cord. 2008;46(10): 684–689. doi: 10.1038/sc.2008.15 18317485

[12] Wright CJ, Colley J, Knudsen K, Kendall E. Housing for People with an Acquired Brain or Spinal Injury: Mapping the Australian Funding Landscape. Int J Environ Res Public Health. 2019;16(16):2822. doi: 10.3390/ijerph16162822 31394883 PMC6721709

[13] Roebuck M. Housing for People with Intellectual Disabilities: A Scoping Review. J Developmental Disabil. 2021;26(2):1–25.

[14] MacLachlan M, Cho HY, Clarke M, et al. Report of the systematic review on potential benefits of accessible home environments for people with functional impairments. In: WHO Housing and Health Guidelines. Geneva: World Health Organization; 2018. Web Annex F. [cited 2024 April 12]. Available from: https://www.ncbi.nlm.nih.gov/books/NBK535292/

[15] Canadian Standards Association. CSA/ASC B652:23, Accessible dwellings. 2023. [cited 2024 April 3]. Available from: https://www.csagroup.org/wp-content/uploads/2430606.pdf

[16] Lindsay S, Fuentes K, Ragunathan S, Li Y, Ross T. Accessible independent housing for people with disabilities: A scoping review of promising practices, policies and interventions. PLoS One, 2024;19(1):e0291228. doi: 10.1371/journal.pone.0291228 38271462 PMC10810508

[17] Gamey J, Terashima M. Accessible Housing in Canada: An overview of policy initiatives and the need for renewed action. Can Plan Pol J. 2023:160–186. doi: 10.24908/cpp-apc.v2023i1.16687

[18] Accelerating Accessibility Coalition. [cited 2024 April 4]. Available from: https://toronto.uli.org/programs/the-accelerating-accessibility-coalition/

[19] Day J, Bobeva M. A generic toolkit for the successful management of Delphi Studies. Elect J Bus Res Method. 2005;3(2):103–117.

[20] Sinha IP, Smyth RL, Williamson PR. Using the Delphi technique to determine which outcomes to measure in clinical trials: recommendations for the future based on a systematic review of existing studies. PLoS Med, 2011;8(1):e1000393. doi: 10.1371/journal.pmed.1000393 21283604 PMC3026691

[21] Hitzig SL, Hunter JP, Ballantyne EC, Katz J, Rapson L, Craven BC, et al. Outcomes and reflections on a consensus-building workshop for developing a spinal cord injury-related chronic pain research agenda. J Spinal Cord Med, 2017. 40(3):258–267. doi: 10.1080/10790268.2015.1136115 26828394 PMC5472012

[22] McDonald L, Hitzig SL, Pillemer KA, Lachs MS, Beaulieu M, Brownell P, et al. Developing a Research Agenda on Resident-to-Resident Aggression: Recommendations From a Consensus Conference. J Elder Abuse Negl, 2015;27(2):146–167. doi: 10.1080/08946566.2014.995869 25836385

[23] Yuzwa K, Co-creating Housing Accessibility Priorities [Internet]. Borealis; 2024. Available from: 10.5683/SP3/WXLFRD

[24] Hsieh HF, Shannon SE. Three approaches to qualitative content analysis. Qual Health Res, 2005;15(9):1277–1288. doi: 10.1177/1049732305276687 16204405

[25] Hsu CC, Sandford BA. The Delphi technique: Making sense of consensus. Pract Assess Res Eval. 2007;12(10):1–8. 10.7275/pdz9-th90

[26] Canada Mortgage and Housing Corporation. Housing Accelerator Fund. [cited 2024 April 11]. Available from: https://www.cmhc-schl.gc.ca/professionals/project-funding-and-mortgage-financing/funding-programs/all-funding-programs/housing-accelerator-fund.

[27] Canada Mortgage and Housing Corporation. Universal Design Guide. [updated 2023 February 23; cited 2024 April 3]. Available from: https://www.cmhc-schl.gc.ca/professionals/industry-innovation-and-leadership/industry-expertise/accessible-adaptable-housing/universal-design-new-multi-unit-residential-buildings/universal-design-guide

[28] Canada Mortgage and Housing Corporation. Accessible housing by design. 2018 [cited 2024 April 3]. Available from: https://publications.gc.ca/collections/collection_2017/schl-cmhc/NH18-24-63J-2016-eng.pdf

[29] Canada Mortgage and Housing Corporation. Case Studies of Sector Initiatives to Meet Accessible Housing Needs. 2020; Ottawa. [cited 2024 April 3]. Available from: https://publications.gc.ca/collections/collection_2021/schl-cmhc/nh18-33/NH18-33-42-2021-eng.pdf

[30] Government of the United Kingdom. Access to and use of buildings—Approved document M. [updated 201 June 7; cited 2024 April 4]. Available from: https://www.gov.uk/government/publications/access-to-and-use-of-buildings-approved-document-m

[31] Livable Housing Australia. Livable Housing Design Guidelines, 4^th^ Edition. 2017. [cited 2024 April 18]. Available from: https://livablehousingaustralia.org.au/wp-content/uploads/2021/02/SLLHA_GuidelinesJuly2017FINAL4.pdf

[32] Australian Building Codes Board. Livable Housing Design Standard. [posted 2023 November 15; cited 2024 April 11]. Available from: https://ncc.abcb.gov.au/resource/standard/livable-housing-design-standard

[33] Taylor E. We Agree, Don’t We? The Delphi Method for Health Environments Research. HERD. 2020 Jan 1;13(1):11–23. doi: 10.1177/1937586719887709 31887097

[34] Powell C. The Delphi technique: myths and realities. J Adv Nurs. 2003;41(4):376–382. doi: 10.1046/j.1365-2648.2003.02537.x 12581103

[35] Government of Canada. Everyone’s business: Accessibility in Canada—Report from the Chief Accessibility Officer, 2023. [posted 2024 February 9; cited 2024 April 18]. Available from: https://www.canada.ca/content/dam/esdc-edsc/documents/corporate/reports/accessibility-disability/everyone-business/6472-ESDC-CAO-report-aoda-en.pdf

[36] Allen S, Resnik L, Roy J. Promoting independence for wheelchair users: the role of home accommodations. Gerontologist. 2006;46(1):115–123. doi: 10.1093/geront/46.1.115 16452291

[37] Habinteg. Living not existing: The economic and social value of wheelchair user homes. 2023. [cited 2024 April 3]. Available from: https://www.habinteg.org.uk/living-not-existing-the-economic-social-value-of-wheelchair-user-homes/

[38] Petersson I, Lilja M, Hammel J, Kottorp A. Impact of home modification services on ability in everyday life for people ageing with disabilities. J Rehabil Med. 2008;40(4): 253–260. doi: 10.2340/16501977-0160 18382820

[39] Ontario Human Rights Commission. Discrimination based on disability and the duty to accommodate: Information for housing providers. [cited 2023 December 7]; Available from: https://www.ohrc.on.ca/en/discrimination-based-disability-and-duty-accommodate-information-housing-providers

[40] Fumarco L. Disability Discrimination in the Italian Rental Housing Market: A Field Experiment with Blind Tenants. Land Economics. 2017;93(4):567–584.

[41] Aranda CL. Targeting Disability Discrimination: Findings and reflection form the national study on housing discrimination against people who are deaf and people who use wheelchairs. Cityscape. 2015;17(3):103–122.

[42] Casson J, Hamdani Y, Dobranowski K, Lake J, McMorris C, Gonazles A, et al. Housing Design and Modifications for Individuals With Intellectual and Developmental Disabilities and Complex Behavioral Needs: Scoping Review. J Policy Pract Intellectual Disabil. 2021;18(3):217–228. 10.1111/jppi.12377

[43] Government of Canada. Charter Statement—Bill C-81: An Act to ensure a barrier-free Canada. 2018. [posted 2018 June 20; updated 2023 June 11; cited 2024 April 11]. Available from: https://www.justice.gc.ca/eng/csj-sjc/pl/charter-charte/c81.html

