# Supplementary material for: Identifying priorities and developing collaborative action plans to improve accessible housing practice, policy, and research in Canada
Source: PLoS One. 2025 Feb 10;20(2):e0318458. doi: 10.1371/journal.pone.0318458 (PMC11809924; doi:10.1371/journal.pone.0318458)
Supplement: S1 Table — (DOCX) [file pone.0318458.s001.docx]

| **Supplemental 1 – Government Relations Issues, Proposed Action(s), and Potential Outcome(s)**   \| **Issues** \| **Proposed Action(s)** \| **Potential Outcome(s)** \| \| --- \| --- \| --- \| \| There is a lack of clear and consistent operational terminology within and across government agency documents (i.e., building codes, national design guides, standards and policies) related to accessible housing.  The length and complexity of  existing standards (i.e., National Standard of Canada CSA/ASC B652:23 Accessible dwellings) creates challenges with interpretation and widespread utility amongst housing developers. \| Work to obtain clarity and consensus on accessible housing terminology across levels of government as well as to standards and guides by providing specific references to what constitutes adaptable or universally designed dwellings.  Enhance standards and guides by providing reasons why specific accessible home features are needed in within these documents. \| Clearer terminology will reduce systemic barriers, confusion, and misinterpretation of policy and legislation that can limit the incorporation and enforcement of key accessibility features in homes.  Providing a rationale for the need of specific accessible home features can serve to support proper implementation by home builders, and address negative attitudinal barriers towards accessible home design.  Shorter documents with clear terminology and simplified standards that align with building code regulations and funding policies would support housing developers to implement the standards in practice with greater ease, consistency, timeliness, and cost-effectiveness. For dwellers, this may lead to homes that are designed to better suit to their accessible housing needs. \| \| Failing to expertly review if initial design, permits, and subsequent inspections of homes are in compliance with accessibility standards can lead to a home that does not meet the end-user needs.  Similarly, if public policies and municipal bylaws are not carefully aligned with the economy of market housing, such as financial penalties that too small and provincial or federal funding that is insufficient, market housing developers will have little motivation to comply with government initiatives, and will choose to pay fines over reducing profits. \| Federal level actions:   1. Acknowledge and take responsible action towards advancement of baseline accessibility in the National Building Code of Canada to reduce discrimination faced by people with disabilities in their right to adequate housing, and improve alignment of Canada’s model building code with the National Housing Strategy Act, the UN Convention on the Rights of Persons with Disabilities, and Canadian human rights codes. 2. Improve data collection on the accessible housing needs for people with disabilities, neurodiversity, older adults, and those in the Deaf and Sight-loss communities, including the use of longitudinal surveys. 3. Improve support of the business supply chain for accessibility related products. 4. Consider implementation of a federal funding program to improve and provide equitable support across the country for accessibility-related modifications, and work to find ways to improve the motivation of property owners and landlords to retro-fit and modify existing homes to meet the accessibility needs of tenants.   Provincial level actions:   1. Hold municipalities accountable for improving the enforcement for developers to build accessible housing, and to find strategies to increase their development. 2. Improve and increase access to funding programs for accessibility-related home modifications, including removing life-time funding caps, increasing funding access to middle-income earners, and expanding available products and features eligible for funding. 3. Provide tax rebate programs for home accessibility related products to support business supply chain. 4. Acknowledge and take responsibility for advancement of accessible housing features in provincial building codes and adoption of new national standards as they arise. 5. Create grants for preferred financing and leasing related to creation of accessible homes. 6. Create cross-ministry programs to improve data collection efforts needed to support understanding of unit costs that impact accessible housing.   Municipal level actions:   1. Hire permanent accessibility coordinator roles who are responsible for providing expertise on City councils in relation to decisions, processes, and policies for accessible housing. 2. Provide city zoning organizational charts and processes to reduce inefficiencies and circular processes that impede the attainment of accessible housing needs to be met, particularly with respect to compliance of current building occupancy classifications and fire code 3. Expedite building permits, zoning applications, and approvals for buildings that prioritize accessible design and retro-fits related to accessibility that are above minimum building code regulations. 4. Create and improve processes for early stage enforcement and auditing of building sites to better enforce accessibility compliance and execution of accessible design within building plans. \| All levels of government would benefit from implementing processes to improve the transfer of information related to accessible housing programs, initiatives, and policies, which could lead to greater incentives and enforcement of regulations and standards related to accessible housing.  Programs that integrate information sharing between the social, health and economic ministries regarding the current lack of availability of accessible housing can lead to a better understanding of its’ importance (e.g., impact or association of inaccessible housing with healthcare unit costs, such as length of hospital stays and alternate level of care provision, long term and residential care admissions, community support and community health care service utilization, etc.  Improved monitoring of the accessible housing stock, improved research and data collection, which could support transparency for responsible use of government funds.  Improved supply chains could lower costs associated with the development of accessible housing for developers and consumers. \| |  |
| --- | --- | --- | --- | --- | --- | --- | --- | --- | --- | --- |
